# Advances and Future Directions of Diagnosis and Management of Pediatric Abusive Head Trauma: A Review of the Literature

**DOI:** 10.3389/fneur.2020.00118

**Published:** 2020-02-20

**Authors:** AM Iqbal O'Meara, Jake Sequeira, Nikki Miller Ferguson

**Affiliations:** Department of Pediatrics, Virginia Commonwealth University, Richmond, VA, United States

**Keywords:** non-accidental head injury, abusive head trauma (AHT), child abuse, TBI, children, intimate partner violence (IPV), subdural hematoma (SDH), inflicted brain injury

## Abstract

Abusive head trauma (AHT) is broadly defined as injury of the skull and intracranial contents as a result of perpetrator-inflicted force and represents a persistent and significant disease burden in children under the age of 4 years. When compared to age-matched controls with typically single occurrence accidental traumatic brain injury (TBI), mortality after AHT is disproportionately high and likely attributable to key differences between injury phenotypes. This article aims to review the epidemiology of AHT, summarize the current state of AHT diagnosis, treatment, and prevention as well as areas for future directions of study. Despite neuroimaging advances and an evolved understanding of AHT, early identification remains a challenge for contemporary clinicians. As such, the reported incidence of 10–30 per 100,000 infants per year may be a considerable underestimate that has not significantly decreased over the past several decades despite social campaigns for public education such as “Never Shake a Baby.” This may reflect caregivers in crisis for whom education is not sufficient without support and intervention, or dangerous environments in which other family members are at risk in addition to the child. Acute management specific to AHT has not advanced beyond usual supportive care for childhood TBI, and prevention and early recognition remain crucial. Moreover, AHT is frequently excluded from studies of childhood TBI, which limits the precise translation of important brain injury research to this population. Repeated injury, antecedent abuse or neglect, delayed medical attention, and high rates of apnea and seizures on presentation are important variables to be considered. More research, including AHT inclusion in childhood TBI studies with comparisons to age-matched controls, and translational models with clinical fidelity are needed to better elucidate the pathophysiology of AHT and inform both clinical care and the development of targeted therapies. Clinical prediction rules, biomarkers, and imaging modalities hold promise, though these have largely been developed and validated in patients after clinically evident AHT has already occurred. Nevertheless, recognition of warning signs and intervention before irreversible harm occurs remains the current best strategy for medical professionals to protect vulnerable infants and toddlers.

## Introduction

As defined by the American Academy of Pediatrics (AAP), abusive head trauma (AHT) is a “well-recognized constellation of brain injuries caused by the directed application of force (shaking or direct impact) to an infant or young child, resulting in physical injury to the head and/or its contents.” (1, 2). The focus of this article is to review the advances and future directions in the diagnosis, treatment, and prevention of pediatric abusive head trauma. AHT encompasses a range of injury mechanisms and clinical outcomes, from subtle presentations requiring a high index of clinical suspicion to moribund infants with lethal injuries. There is ample evidence to support the existence and diagnosis of AHT, from clinical observation and study, multi-specialty expert consensus, multi-species animal models, and perpetrator confession (3–10). A 2009 policy statement from the AAP recommended that pediatricians use AHT rather than a term that implies a single injury mechanism, such as the previous moniker “shaken baby syndrome.” (2). AHT is characterized by an aggregate of physical, radiographic, and laboratory evidence that cannot be explained by the provided history or is incongruent with the developmental stage of the child. Children suffering from AHT generally benefit from advances in traumatic brain injury (TBI) care, but there remain disproportionate mortality and poor outcomes in survivors of AHT as compared to accidental traumatic brain injury (TBI), making prevention and early identification paramount (11–14).

While there remains some controversy in the legal community surrounding the diagnosis of AHT and the intensity and/or mechanical forces that are necessary to cause the spectrum of associated injuries, there is no scientific controversy regarding the clinical diagnosis of AHT (15–20). Defense strategies have historically relied upon undermining the diagnosis of AHT and “inappropriate use of scientifically unsupported alternative theories.” (20). Our understanding of AHT has evolved and coincided with developments in the field of radiology that have facilitated the identification of hemorrhages, parenchymal injuries, and fractures that could only be attributed to physical abuse. Formally named “The Battered Child Syndrome” by C. Henry Kempe in 1962, the term described a series of symptoms and findings that should prompt practitioners to suspect harm by caregivers (21). Further research in the 1970s posed shaking or whiplash injury as an important mechanism of subdural hemorrhage (SDH) in these patients (22, 23). There is contemporary literature as well as perpetrator confession to support that not only is whiplash-shaking alone sufficient to cause SDH, but retinal hemorrhages as well (5–7, 24). Acceleration-deceleration impact and rotational force injuries are also better understood in recent years, with data indicating that comparatively mild non-accidental head trauma can result in significant injury, particularly when repetitive, or when medical care is delayed (25).

## Epidemiology of AHT

### Incidence and Risk Factors

Current estimates place the incidence of abusive head trauma between 10 and 30 per 100,000 infants per year, with the highest incidence in the first 2 years of life (26–28). Historically, there has been a male predominance in diagnosed AHT cases, but a few recent studies suggest that females may be equally represented, if not more (29, 30). Low socioeconomic status and domestic violence have been cited as risk factors, and recent epidemiologic studies have further identified young maternal age, male caregivers, and caregiver substance abuse or mental health disorders as additional risk factors for AHT (8, 26, 31). This data is likely incomplete, as there is not a “gold standard” diagnostic for AHT. Additionally, under-reporting and delayed recognition remain significant issues.

### Impact/Outcome

AHT presents a significant chronic societal burden, not only in direct costs but also in lost potential and productivity. Beyond the already exceedingly high cost of treating a child with AHT from injury through convalescence lies the debilitating strain placed on families and society (32). Miller et al. estimated the overall impact of the estimated 4824 AHT (fatal and non-fatal) cases in 2010 at ~$13.5 billion, factoring in medical expenses, long-term care, and social intervention (child protective services and criminal justice costs). A large portion of this estimated cost comes from the work loss cost. Interestingly, even a “mild” case with a reasonable outcome has an average estimated loss of 15% of health-related quality of life (33). Attempts to objectively measure the degree of impairment have met with difficulty, as this can be a fairly subjective term. However, using self-report surveys of disability like the Health Utilities Index (HUI), it is estimated that over the lifespan of a survivor of AHT, overall quality of life may range from 80% for mild AHT, all the way down to 40% for severe AHT survivors (HUI score represents percentage of quality of life someone has compared to person in perfect health) (34). Disease burden in terms of disability has also proven to be extremely problematic in AHT. Disability-adjusted life-years (DALYs) are calculated by summing years of productive life that survivors lose to disability plus years lost to premature death. In the case of severe AHT, annual DALY per surviving child averaged 0.555 years, with an estimated average lifetime DALY burden of 24.1 years. Put in perspective, even mild AHT poses a DALY burden that exceeds that of a severe burn (34, 35).

Given perceptions of the neuroplasticity of youth and implied recovery potential, it may be counterintuitive at first that typical brain injury patterns and ultimate outcomes are worse in AHT than those following accidental TBI (such as motor vehicle collisions and witnessed falls) (36–40). When adjusted for age, it has been demonstrated a nearly 10-fold higher incidence of neurosurgical intervention in AHT patients compared to their accidental trauma counterparts (12). Studies indicate mortality rates ranging from 18 to 25% (8, 27, 29, 41). For those that survive, 20–40% will do so with severe disability, defined as gross neurologic impairment requiring full assistance in activities of daily living. For the remainder, longitudinal studies report high rates of neuromotor, psychiatric, and cognitive deficits (31, 37, 38, 42). Poor outcomes are multifactorial, likely attributable to the age and neurodevelopmental state of these patients, chronicity of abuse, the type and timing of injury, as well as delayed presentation leading to additional insults known to worsen outcomes after TBI (43).

### Injury Mechanisms/Pathophysiology

AHT patients tend to experience a high burden of secondary insults before presenting to medical care, including apnea with consequent hypoxemia and hypotension, and seizures (29, 44, 45). Coinciding systemic polytrauma with fractures and intra-abdominal injury can exacerbate marked anemia, coagulopathy, systemic inflammatory responses, and shock. Furthermore, even mild TBI is recognized to cause persistent perturbations in not only cerebrovascular autoregulation, but also autonomic regulation and inflammatory and apoptotic cascades such that every subsequent injury is not simply additive, but the consequences are exponential (46, 47). Vavilala et al. found in a small cohort of AHT that all had impaired cerebral autoregulation, either unilateral or bilateral hemispheric dysfunction (48). Studied most heavily in contact-sport athletes, “second-hit syndrome” is not elucidated in AHT, but given transcranial doppler evidence of altered vascular tone early after acute pediatric TBI, the repetitive concussion of contact-sports presents an interesting parallel in terms of the pathophysiology of neuronal and glial injury with sustained vulnerability and represents an area that might inform further study (49–51).

The immature brain requires an inherently different balance of neurotransmission, blood flow, and energy requirements that, while important for early neurodevelopment, may predispose to a poorer injury phenotype (52). There are two major pathologic mechanisms for secondary damage and cell death after trauma that have been identified- excitotoxicity and apoptosis. An overabundance of actively developing and immature dendrites and synapses is vital in early childhood but may potentiate excitotoxicity which occurs within the first few hours after the primary insult. Additionally, microglia play an important role in regulating dendritosynaptogenesis, and may be primed in a manner that exacerbates the neuroinflammatory response after injury. Animal models have shown that apoptosis appears to be the more devastating event in producing significantly higher rates of cell death than excitotoxicity in immature rodents (53, 54). These studies also have shown an age-dependent effect on apoptotic cell death, with younger rodents (3, 7 day old) demonstrating increased vulnerability for trauma-induced apoptosis. An intrinsic need to cull extraneous neurons and synapses through apoptosis and pruning during normal developmental remodeling appear to negatively sway the cell survival balance after injury, as interestingly the highest proportion of apoptotic cells after trauma were found in areas that had the highest densities of cells undergoing physiologic apoptosis in sham animals (53, 54). If the injury involves areas of the brain with a narrow developmental window or interdependent connectivity with non-contiguous regions, functional outcomes in survivors can be impacted dramatically. Additionally, trauma during this period may also interfere with ongoing developmental events such as neuronal migration, and axonal and dendritic growth by altering the proteins that guide these processes (55).

## Diagnosing AHT

### Clinical Features

Presenting history is important in identifying AHT, as is a physical examination with a high index of suspicion when indicators are present. While caregiver histories are frequently not forthcoming, the incongruity of an explanation with presentation is a hallmark of AHT. In the prehospital phase of care, children with AHT are nearly twice as likely to have been transported from home, often by private vehicle with little to no resuscitation (29). Apnea has been shown in several studies to be significantly associated with AHT as compared to accidental TBI, as are seizures, with studies finding 28–50% of AHT with seizures upon presentation (29, 44, 45). History of developmental or growth delay should raise concern, as should a history of vomiting (without diarrhea), increased head circumference, and/or excessive irritability (44). Rib, long bone, and complex skull fractures support a diagnosis of AHT, and retinal and subdural hemorrhages have historically been the most relied on indicators of an abusive injury. While not required for diagnosis, retinal hemorrhages have been reported in up to 85% of AHT victims, and tend to be diffuse and bilateral, involving all layers of the retina (56). Subdural hemorrhages have been reported in >70% of AHT victims (45, 57, 58). Specific neuroimaging patterns were further described by Kemp et al. in 2011: multiple SDH over the convexity, interhemispheric hemorrhages, posterior fossa SDH, hypoxic-ischemic injury (HII), and cerebral edema were significantly associated with AHT (59). Chronic SDH appears to be specific for AHT, if not particularly sensitive, with less than half of identified AHT cases presenting with chronic SDH (vs. the far more common acute SDH) (60).

### Imaging

**Figure 1:**
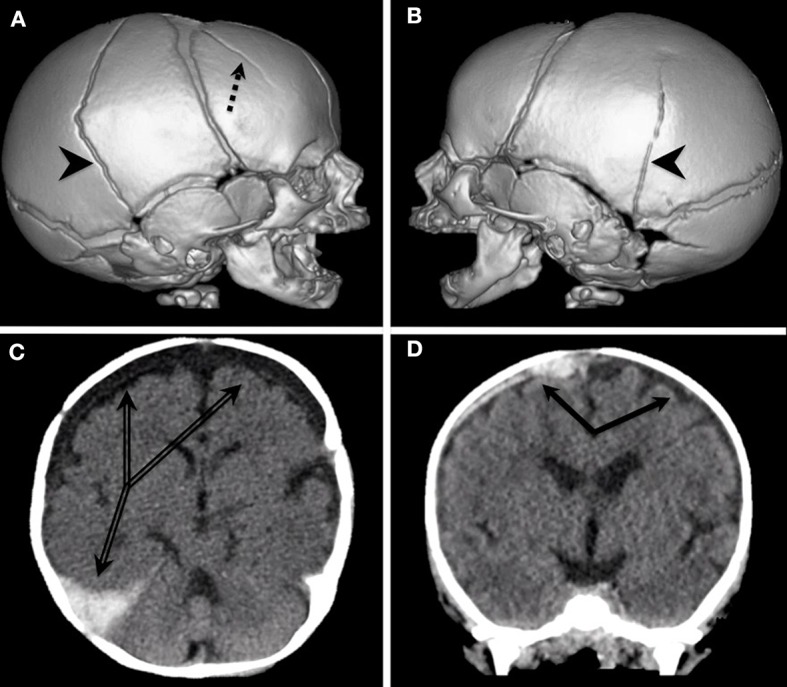
**(A,B)** Multiple, bilateral skull fractures as a result of AHT depicted with 3D calvarial reconstruction. Right posterior temporal fracture extending obliquely over the vertex to the left posterior temporal region (arrowheads) with additional fracture anterior to the right coronal suture (dashed arrow). **(C,D)** Mixed density subdural collections resulting from AHT. Image C demonstrates neomembranes in chronic subdural hygromas over the bifrontotemporal convexities with a newer hyperdensity in the right temporo-occipital convexity extending into the cerebral falx (double line arrows).

Non-contrast head computed tomography (CT) is generally the first imaging modality for acute traumatic or unexplained encephalopathy, as it rapidly informs the need for urgent neurosurgical interventions such as hematoma evacuation or cerebrospinal fluid (CSF) diversion. Ideally, initial head CT should include 3D calvarial reconstruction for the accurate representation of skull fractures, as depicted in Figures 1A, B (61). Thereafter, additional complementary magnetic resonance imaging (MRI) is more sensitive for parenchymal injuries, diffuse axonal injury, injury to bridging veins, and early evidence of HII and cerebral edema, all of which can be seen in AHT and contribute to high morbidity and mortality. Additionally, MRI may be used to differentiate subdural hemorrhage from benign enlargement of the subarachnoid space (BESS) as chronic SDH may be difficult to distinguish on CT. Neuroimaging should not be solely relied upon for precisely pinpointing the age of SDH due to variability of hematoma appearance and evolution when combined with less dense CSF, a consequence of traumatic violation of the arachnoid membrane and leakage of CSF into the subdural space (62). Mixed-density SDH, as shown in Figures 1C,D, is more frequently observed in AHT than simple hyperdensity typical of acute hematoma blood products (58 vs. 28%, respectively, with 14% appearing hypodense in a series of 105 confirmed AHT) (63). Additionally, mixed-density SDH is more frequently observed in AHT than accidental TBI (63). This may be related to the presence of co-mingled CSF, but could also result from an antecedent subacute injury or acute-on-chronic collection. It can be challenging to differentiate on a single imaging study in isolation, as hyperdense blood product resolution varies in its timeline from 48 h to 40 days (9). Heterogeneous mixed-density SDH with distinct regional differences (hypodense in one area and hyperdense in another) may or may not be suggestive of separate injury events, but the development of intradural neomembranes is consistent with an injury at least 10 days to several weeks old, as seen in Figure 1C. With caveats regarding the variability in SDH appearance and hematoma evolution, there may be a role for neuroimaging in establishing an injury timeline when combined with other clinical, historical, and radiographic findings, particularly in the exclusion of other cranial lesions or fractures having occurred in the period suggested by a witness, and/or if serial neuroimaging is obtained (9, 63, 64). The recommended work-up of AHT includes a full skeletal survey, which can support both diagnosis and chronicity of abuse with systemic fractures in varying stages of healing.

Spinal imaging of soft tissues with MRI is more recently recognized to support the diagnosis of AHT, and should be strongly considered for inclusion when a brain MRI is obtained in suspected AHT, or when spinal injury is suspected (9). Compared to adults, children have disproportionately large heads, supported on relatively weak necks. Given this physiology and the prevalence of shaking injury, cervical injuries are much more common than previously thought, but until recently clinicians lacked the imaging modalities necessary to make an early diagnosis. Studies in the 1980s and 1990s found a significant incidence of cervical injury in confirmed cases of AHT, however, these findings were made on autopsy (65, 66). In the last decade, the advent of advanced imaging with MRI has been used to estimate the incidence of cervical spine injury with AHT at anywhere from 15 to 46%, with over 80% incidence in those patients with AHT involving bilateral HII (9, 67–69). Interestingly, this type of high cervical injury may torque, stretch, or otherwise injure the brainstem, inducing apnea in an injury pattern that may not only be peculiar to AHT, but may explain differences in clinical presentation and outcomes given the strong association of cervical spine injury and HII (69, 70).

### Clinical Prediction Rules

Clinical prediction rules (CPRs) for AHT are intended to facilitate early recognition of abuse as the proximate cause of intracranial injury so that additional confirmatory workup can be pursued and other injuries identified. Ideally, CPRs should also help avoid unnecessary testing and prevent unwarranted accusation of a caregiver. The currently published CPRs for AHT “aids or prompts” the clinician to “seek further information, investigation and assessment” order for them to diagnose AHT (71). CPRs do not diagnose AHT by themselves and should supplant rather than replace clinical acumen. Importantly, each of the CPRs that have been validated are for specific populations in specific stages of their workup, none of which are in a primary care/outpatient setting. Concern has been raised regarding the potential of a false sense of security, especially in clinicians who are not as familiar/have less experience with AHT, if a CPR gives a low probability of AHT (72). These tools are designed to be used in conjunction with a complete history and physical exam as well as clinician expertise and judgement in order to more robustly approach decision making in AHT evaluations.

Important attempts in recent years to develop CPRs have seen the addition of historical elements, clinical, and imaging findings in order to more accurately identify AHT (Table 1). In 2013, the Pediatric Brain Injury Network (PediBIRN) derived a CPR for patients admitted to Pediatric Intensive Care Unit (PICU) based on acute respiratory compromise before admission; the presence of ear, torso, or neck bruising; bilateral, or interhemispheric SDH; and any skull fractures other than an isolated, unilateral, non-diastatic, linear, parietal fracture (73). With just these four criteria, a validation study found that the PediBIRN score identified 98% of PICU patients ultimately diagnosed with AHT (74). Recently, PediBIRN was externally validated in the intended PICU setting as well as in all children <3 years old admitted with imaging-confirmed intracranial injury in Australia and New Zealand. Similar to the original validation study, PediBIRN CPR was highly sensitive with 96% sensitivity among all admitted patients, and 100% sensitive for patients admitted to the PICU (75). Although not yet externally validated, an update to the PediBIRN in 2019 saw the creation of the PediBIRN-7, which includes results of the AHT workup (imaging- skeletal survey and neuroimaging; retinal exam) to predict probability of AHT in order to further inform a clinician's diagnosis (76). The Predicting Abusive Head Trauma (PredAHT) CPR used 6 clinical indicators and found that when ≥3 of these are present, the estimated probability for AHT is >81.5% (77, 78). The sensitivity of the tool based on a 50% probability cut-off is 72.3% and specificity of 85.7% (77, 78). PredAHT-2 was updated to account for missing data, as well as externally validated in an Australian/New Zealand population (79). The Pittsburgh Infant Brain Injury Score (PIBIS) was developed by Berger et al. to guide the decision-making process for neuroimaging in otherwise healthy infants presenting to the ED at risk for AHT given symptoms that could be attributed to intracranial pathology in the absence of a trauma history. The score is based on the presence of abnormal skin exam (bruising), age > 3 months, head circumference > 85th percentile, and serum hemoglobin <11.2 g/dL. Using these data, validation studies identified a sensitivity of 93.3%, a specificity of 53%, and a positive predictive value of 39% for abnormal neuroimaging (80). Research is also ongoing to develop CPRs that will detect AHT even in the case of equivocal history or exam findings; in 2017, Berger et al. introduced the Biomarker of Infant Brain Injury Score (BIBIS), a panel composed of three serum biomarkers and serum hemoglobin, which identified 89.3% of patients with acute intracranial hemorrhage, with a 95.6% negative prediction value (81). By necessity, screening CPRs have high sensitivity with the trade-off of lower specificity. PediBIRN is excellent for prompting the consideration of abuse in young brain-injured children admitted to the PICU, and PIBIS captured a very high rate of acute intracranial pathology on neuroimaging in patients in the ED that otherwise might not be obtained. PredAHT was much more specific than either PediBIRN or PIBIS in patients admitted to the hospital, and may be useful not only as an independent CPR, but in conjunction with PediBIRN and/or PIBIS may guide investigative work-up (71).

**Table 1:** Externally validated CPRs for prompting the recognition and/or consideration of AHT as the proximate cause of acute intracranial injury in infants and toddlers.

	**Predicting Abusive Head Trauma (PredAHT)**	**Pittsburgh Infant Brain Injury Score (PIBIS)**	**Biomarkers for Infant Brain Injury Score (BIBIS)**	**Pediatric Brain Injury Network (PediBIRN-4)**	**7-Variable Clinical Prediction Rule (PediBIRN-7)**
Use	Estimating AHT probability in a brain injured infant or toddler	Screening high risk infants and toddlers for neuroimaging in the absence of a trauma history	Screening high risk infants for neuroimaging in the absence of a trauma history	Estimating AHT probability in a brain injured infant or toddler	Estimating AHT probability in a brain injured infant or toddler
Variables	1) Apnea 2) Head or neck bruising 3) Seizure 4) Rib fracture 5) Long bone fracture 6) Retinal hemorrhages	1) Age > 3 months (1 point) 2) Bruising on skin exam (2 points) 3) Head circumference >85th percentile (1 point) 4) Serum hemoglobin <11.3 g/dL (1 point)	Serum biomarkers: 1) Matrix metallopeptidase-9 2) Neuron-specific enolase 3) Vascular cellular adhesion molecule-1 4) Hemoglobin	1) Respiratory compromise 2) Bruising of ear, neck, or torso 3) Bilateral or interhemispheric subdural(s) hemorrhage or fluid collection(s) 4) Skull fracture other than simple, linear parietal skull fracture	1) Respiratory compromise 2) Bruising of ear, neck, or torso 3) Bilateral or interhemispheric subdural(s) hemorrhage or fluid collection(s) 4) Skullfractureother than simple, linear parietal skull fracture 5) Positive skeletal survey^*^ 6) Positive ophthalmological exam^**^ 7) Brain hypoxia, ischemia, or swelling
Clinical Scenario	<3 years of age admitted with intracranial injury found on neuroimaging	Well-appearing, afebrile infants without a history of head trauma presenting with: 1) Apnea/apparent life-threatening event 2) Vomiting without diarrhea 3) Seizures or seizure-like activity 4) Soft tissue swelling of scalp 5) Bruising 6) Other nonspecific neurologic symptom such as lethargy, fussiness, poor feeding	Well-appearing, afebrile infants without a history of head trauma presenting with: 1) Apnea/apparent life-threatening event 2) Vomiting without diarrhea 3) Seizures or seizure-like activity 4) Soft tissue swelling of scalp 5) Bruising 6) Other nonspecific neurologic symptom such as lethargy, fussiness, poor feeding	<3 years of age admitted to pediatric intensive care unit with intracranial injury found on neuroimaging	<3 years of age admitted to pediatric intensive care unit with intracranial injury found on neuroimaging
Sensitivity/Specificity during Validation	With a 50% probability cutoff, 72% sensitivity and 86% specificity	At a score of ≥ 2, 93% sensitivity, 53% specificity for abnormal neuroimaging (traumatic or otherwise)	With a cutoff of 0.182 when AUC 0.91, 89.3% sensitivity and 48% specificity for acute intracranial hemorrhage	96% sensitivity and 46% specificity in intensive care patients	With a 50% probability cutoff, 73% sensitivity and 87% specificity in intensive care patients (derivation, not validation study)

* Positive skeletal survey: classic metaphyseal fractures, epiphyseal separation(s), fracture(s) involving the rib(s), digit(s), scapula, sternum, or spinous process(es), or vertebral body fracture or dislocation.

** *Positive ophthalmologic exam: retinoschisis or retinal hemorrhages described as dense, extensive, and/or extending to the periphery (oro serrata)*.

### Management of AHT

AHT is a heterogeneous insult, and as such, management occupies a broad spectrum of tools and therapies. Initial care in the pediatric patient with AHT is directed toward stabilization of the airway, support of oxygenation, ventilation, hemodynamics, and mitigation of intracranial pathology. Children with AHT should be evaluated at a level 1 Pediatric Trauma center with access to *pediatric* specialists such as neurosurgery, trauma surgery, neurology/epileptologist, child abuse pediatrician, and intensivists. Mild injuries may simply require supportive care; keeping hemodynamics and physicochemical milieu in a normal range, coupled with simple maneuvers such as keeping patients partially upright in bed with the head positioned midline. For more severe injury, the Pediatric Severe TBI Guidelines (severe TBI defined by a GCS<9) suggest the use of invasive intracranial pressure (ICP) monitoring with subsequent ICP-driven management for improved outcomes (82), although there remain differing practices and debate regarding the utility of ICP monitoring in infants with open fontanelles. While the Guidelines do not specifically address the AHT population separately, the authors do state “the presence of open fontanelles and/or sutures in an infant with severe TBI does not preclude the development of intracranial hypertension or negate the utility of ICP monitoring” (83).

### Acute Management

Intracranial hemorrhage and edema being commonly seen in AHT, management of the resultant increased ICP has become one of the primary goals of acute treatment, following the *Guidelines for the Management of Pediatric TBI, third edition* (82). However, the exact goals of management remain unclear. In the specific context of AHT, persistent increased ICP > 20 mmHg and cerebral perfusion pressure (CPP) <45 mmHg appear to correlate with worsened outcome (21). What remains to be seen is whether or not more aggressive (lower ICP, higher CPP) goals will add additional therapeutic benefit. Findings by Jha et al. using longitudinally monitored ICP trajectories in adults may provide new insights into AHT interventions and outcomes; when continuously plotted out, patients with persistently low ICP trajectories had unfavorable outcomes that were only slightly better than the patients with severe, persistent intracranial hypertension. Strangely enough, the patients with higher ICP (~14 mmHg) and frequent spikes had the best outcomes, a finding that may indicate that the practice of driving ICP under 20 mmHg for all patients may be too simplistic (84). Given the highly heterogeneous nature of AHT, it is entirely possible that the optimal intervention is one in which the clinician allows for some of the natural evolution of AHT to take place in order to better phenotype the injury and appropriately treat.

HII is prevalent in AHT, and may be related to apnea-associated hypoxemia and hypotension, relative ischemia from early posttraumatic seizures, cerebral edema and vascular compromise, or anemia and hypotension after significant intracranial or systemic hemorrhage (4, 85). Seizure severity seems tied to the degree of HII, and evidence of HII on MRI may evolve over time (86). In a recent study of the first 200 patients of the ADAPT trial, there was significantly more reported or observed apnea in the AHT cohort compared to accidental TBI, despite no differences in rates of documented hypoxemia or hypotension during prehospital care (29). Interestingly, the criteria for hypoxia/hypotension in this study were quite conservative and may have missed clinically relevant episodes of both. Furthermore, AHT patients were more likely to arrive via private vehicle without trained prehospital care providers and consequently, hypoxemia and hypotension were likely unrecognized or undocumented. As with all TBI, hypoxemia and hypotension are key factors linked to poor outcome, and prompt recognition and correction of these perturbations is essential. Unfortunately, the typical presentation of AHT often delays proper resuscitation.

In addition to HII, age (<2 year) and severity of injury (SDH or GCS<8) are strong predictors of seizures after AHT (87, 88). Primary brain injury is exacerbated by seizure-related excitotoxicity and metabolic stress, and early and effective treatment is necessary. There is a risk of under-recognition, as seizures may be subclinical; Hasbani et al. demonstrated in a study of 32 children with AHT that over half of the children monitored on EEG were found to have seizures. Of these children, 67% had subclinical seizures that would have otherwise gone undetected without EEG (89). As such, there is a role for continuous EEG monitoring in AHT to detect subclinical seizures, particularly in the case of coma after resuscitation, or if the child has received sedation or neuromuscular blockade (90).

Given the potentially devastating effects of seizures after TBI, much effort has been expended in developing therapeutic strategies to mitigate epileptiform activity. The current body of literature indicates that early posttraumatic seizures (EPTS; defined as seizures occurring within 7d of injury) are more common in children vs. their adult counterparts who tend to develop late posttraumatic seizures (LPTS) (91). Retrospective studies demonstrate that upwards of 50% of children with severe AHT experience EPTS without antiepileptic drug (AED) prophylaxis, compared with only 15% who developed EPTS when prophylaxed with phenytoin (92, 93). As such, current guidelines suggest prophylactic treatment for early seizure, but have removed phenytoin from the previous Level III recommendation stating “insufficient evidence to recommend levetiracetam over phenytoin.” (82, 94). Alternative AEDs have been investigated in recent years, with levetiracetam being the most common, citing a better side effect profile. However, a recent study failed to show any benefit over phenytoin, in fact showing that levetiracetam may be less efficacious as prophylaxis in pediatric TBI (92).

While the control of post-AHT seizures is a mainstay of therapy, it is unclear whether or not long term functional outcomes are changed by rigorous seizure control. In adults, posttraumatic seizures are associated with a worse long-term functional outcome, and early prophylaxis and aggressive seizure treatment is standard for both adults and children (91). While data indicates that prophylactic AED therapy may prevent EPTS, it also demonstrates no benefit in the reduction of late posttraumatic seizures (LPTS) or posttraumatic epilepsy (95). Indeed, currently, no pharmacologic therapy is as of yet established to prevent the development of LPTS, or to reduce mortality (96, 97).

### Post-Acute Management

Care after discharge is equally nebulous. It has been shown that AHT patients make significant functional gain and do benefit from being discharged to an inpatient rehabilitation center (98). Unfortunately, there is a dearth of pediatric rehabilitation facilities in the US requiring patients at times to go far from their home, even out of state for some, or to be discharged home with the hope of receiving adequate outpatient rehabilitation. There also remains the issue of an AHT victim returning home to an unstable or unsafe living environment. The role of stress in the developing pediatric brain is ill-defined, but studies point to the detrimental effects of stress on the immune and inflammatory response (99, 100). Even something as seemingly simple as separation from a caregiver has been demonstrated to upregulate inflammatory factors in animal models, and in human children, domestic stress and violence has been linked to asthma (100–106). More worryingly, current understanding of neuroinflammatory pathways would seem to point toward a discrete role for social stress in potentiating future neurocognitive disabilities in the pediatric patient. Already implicated in anxiety and depression in adults, in the pediatric patient, there may be an increased risk for behavioral dysfunction, if not outright cognitive delay, as rodent models are beginning to suggest that the stress response may be implicated in neural pruning in the hippocampus and amygdala (107, 108). As of now, no studies have clearly defined a relationship to stress and long term neurocognitive outcomes, particularly in the context of head trauma.

## Prevention

Given the insidious nature of the disease process, and the difficulties inherent to treatment and recovery, the prevention of AHT is the current best strategy available to clinicians. As AHT is by definition an injury perpetrated out of social dysfunction, these preventative measures have been based almost entirely around caregiver education and social support. The current body of literature has identified two primary areas to reducing AHT: parental education about infant crying and risks of shaking a baby (109). Understanding of caregiver personal and social resources is key in developing targeted strategies to ensure safe and effective care. Current psychological therapy geared toward generating this mindset focuses on fostering emotional regulation; articulation of caregivers' particular strengths, empathy, power-sharing in the child-raising unit, and impulse control are core components of therapy and education (110). Such programs have met with mixed success, with some showing up to 35% reduction in AHT admissions, while others showing no reduction in AHT rates (111–113).

The role of the pediatrician is 2-fold, both as a primary clinician responsible for the detection of early symptoms concerning for abuse and as an educator to caregivers. It is important for clinicians to be educated in recognizing the sometimes subtle signs of non-accidental injuries such as bruising or fractures in non-cruising infants, vomiting without diarrhea, lethargy, poor oral intake, or injuries without adequate trauma history (114, 115). Studies have demonstrated frequently missed opportunities to diagnose sentinel abusive injuries in children who were later diagnosed with AHT (116–118). Unfortunately, as shown in a recent study by Letson et al. there has not been a significant improvement in the rate of missed sentinel abuse events over the last two decades (117). Setting expectations with young and first-time parents regarding what constitutes normal crying patterns by primary providers, and what constitutes normal infant interactions has a significant impact on caregiver satisfaction. Shaking as a behavioral control technique has a particularly insidious element; for a parent that doesn't know better, shaking quiets a crying infant and results in a positive reinforcement loop (119). This is evidenced by the fact that in situations where parents have admitted to shaking their infants, shaking was repeated in 55% of the cases (on average 10 times), occurring daily for several weeks in 20% of the cases, and was repeated because it stopped crying in all cases (7). Methods to mitigate crying in a healthy way range from soothing techniques, to simply educating parents regarding the normal crying patterns of infants, and the dangers of shaking an infant.

There is an association between intimate partner violence (IPV) and child abuse in families (120–124). In one study, 59% of children who were evaluated by child abuse providers after IPV exposure were found to have an injury. Of those, 24.6% had internal injuries including fractures, intracranial, or intra-abdominal injury, with almost all of these children being <1 year. Of those found to have injuries upon evaluation, 44.4% had either no report of direct injury or a mechanism that did not explain the injury. Even more concerning, several of these patients did not have any physical exam findings to suggest their internal injuries (122). This and other studies highlight the need for greater recognition of children in an IPV environment. As pediatricians specialized in child abuse are limited resources, there needs to be a wider net in the medical community who are educated on the risk factors, signs, and symptoms of AHT. This includes adult providers who may be seeing a patient with IPV concerns which should prompt the question “Where are your children? Are they at home with this partner?” IPV has been associated with AHT and a recent meta-analysis determined that the odds of child abuse in a family with reported IPV was 3.64 (98). Police, EMS, CPS, and other such first responders should be educated regarding this association and trained to ask about any children in the home when responding to IPV incidents. If there are children, especially those <1 year of age, they should be seen by a medical provider trained to evaluate for child abuse.

## Future Directions

Prevention and detection of AHT remains the first line in future management. Studies examining local and statewide educational programs have shown variable results in attempts to reduce AHT incidence, and have so far failed to identify which key components are most effective (111, 112). With the continuation of widespread education, it will be crucial to determine which strategies are most effective. Continued refinement and testing of CPRs will similarly be vital in the future management of AHT, as earlier detection of injury will be key to minimizing morbidity and mortality. Future refinements of CPRs would ideally provide clinicians with powerful tools to increase their confidence in diagnosis and standardize the process (125).

Disproportionately poor outcomes in the moderate to severe spectrum of AHT when compared to age-matched accidental TBI are multifactorial. Delay in medical care, repeated injury, prolonged seizures on presentation, and apnea with its consequences certainly play critical roles, and highlight the need for education, prevention, and intervention before significant injury occurs. A pre-injury factor which warrants investigation is exposure to abusive or neglectful environments. Repeated, unpredictable stress in the developing mammalian brain has been observed to potentiate immunomodulation in animal models, and may produce a unique pro-inflammatory phenotype with a lowered seizure threshold in response to brain injury (100). This is of particular interest in AHT, where victims may spend a great deal of time in abusive households before being identified, or may return to high-stress households during convalescence following treatment.

There remains a great deal of variation in the acute care of TBI, let alone AHT. Lack of clear consensus regarding goals of treatment and outcome metrics contribute to a lack of standardized intervention after AHT is diagnosed. The typical example of this heterogeneity is that ICP monitoring is not standardly employed for these infants, despite evidence that centers with standardized ICP monitoring for pediatric TBI have improved patient outcomes (126). This may reflect improved ICP-guided management, or may indicate that centers with greater expertise are more likely to invasively monitor these patients. While there is a growing body of literature on AHT as a distinct form of TBI, there remains a dearth of evidence for AHT-targeted therapies. These patients have often been excluded from pediatric TBI studies, making it difficult to extrapolate lessons learned from accidental TBI to the AHT population. Inclusion of these patients in larger, multi-center studies of pediatric TBI, with subanalyses of the AHT cohort against age-matched controls, will move the care of these patients forward and identify important differences for focused study.

## Author Contributions

AMI and NM contributed to conception of the manuscript and wrote sections of the manuscript and revised it critically. JS wrote the first draft of the manuscript. All authors contributed to manuscript revision, read, and approved the submitted version.

### Conflict of Interest

The authors declare that the research was conducted in the absence of any commercial or financial relationships that could be construed as a potential conflict of interest.
